# Supplementary material for: Feasibility of Longitudinal Relaxation Rate Mapping with Non-Cartesian Sampling and Compressed Sensing on a 1.5T MR-Linac
Source: medRxiv. 2025 Jul 29:2025.07.28.25332213. Preprint. [Version 1] doi: 10.1101/2025.07.28.25332213 (PMC12324627; doi:10.1101/2025.07.28.25332213)
Supplement: 1 [file NIHPP2025.07.28.25332213V1-supplement-1.pdf]

medRxiv preprint doi: https://doi.org/10.1101/2025.07.28.25332213; this version posted July 29, 2025. The copyright holder for this preprint (which was not certified by peer review) is the author/funder, who has granted medRxiv a license to display the preprint in perpetuity.
It is made available under a CC-BY 4.0 International license .

6. Supplementary Materials

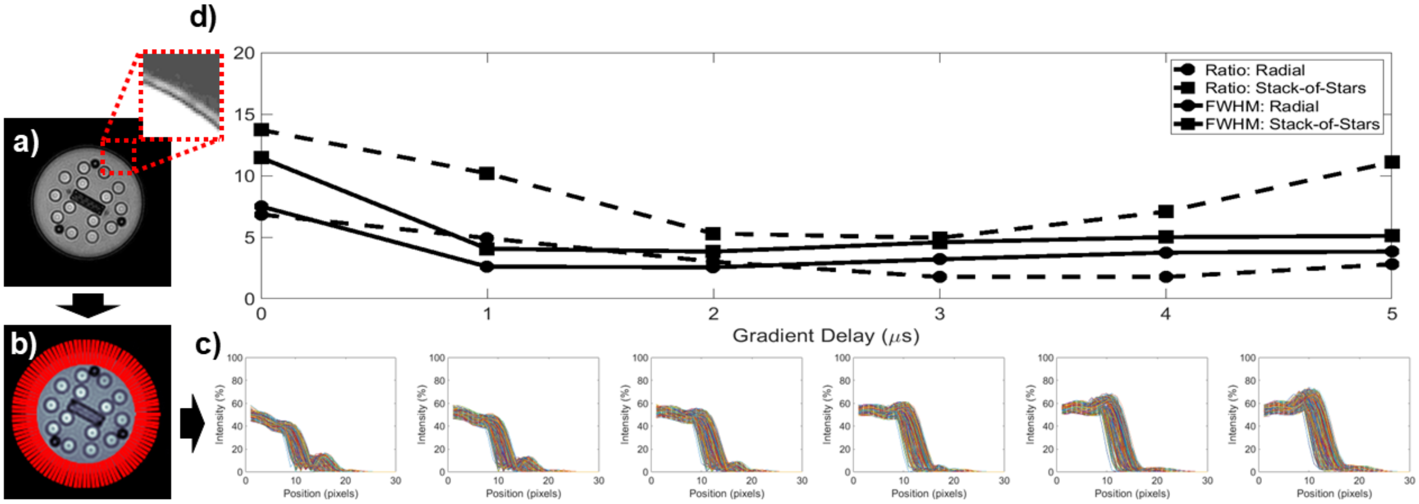

Figure S1: Overview of analysis for gradient delay time optimization. a) Ghosting artifact at the edge of the phantom, b) A set of radial spokes placed every 1° to analyze line profiles, c) Intensity line profiles for each radial spoke, d) Computed ratio and FWHM for both the half-spoke radial and stack-of-stars sequences.

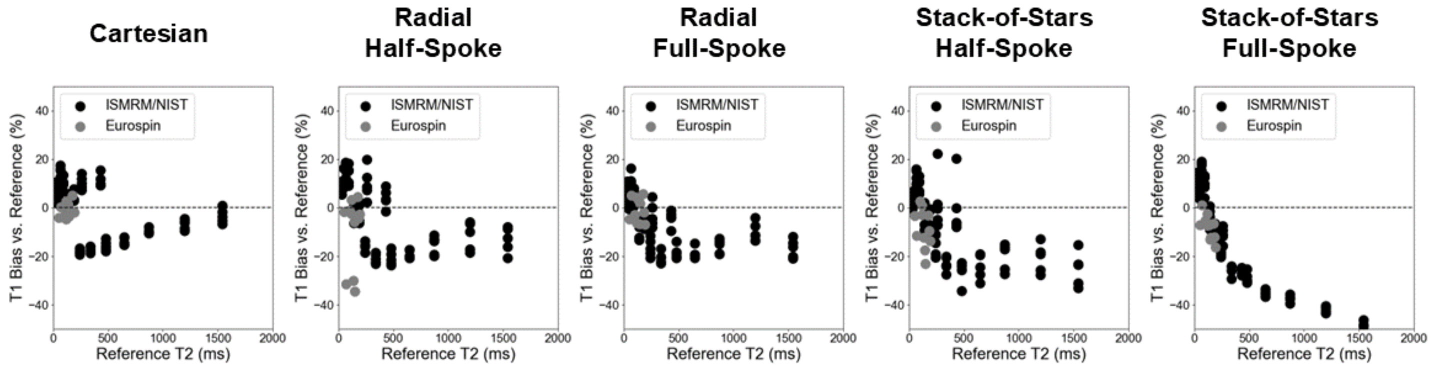

Figure S2: T1 bias as a function of reference T2 values for all vials in the ISMRM/NIST and Eurospin phantom. Note general trend in increase in bias due to insufficient spoiling and signal recovery in vials with high T1 and T2 values.

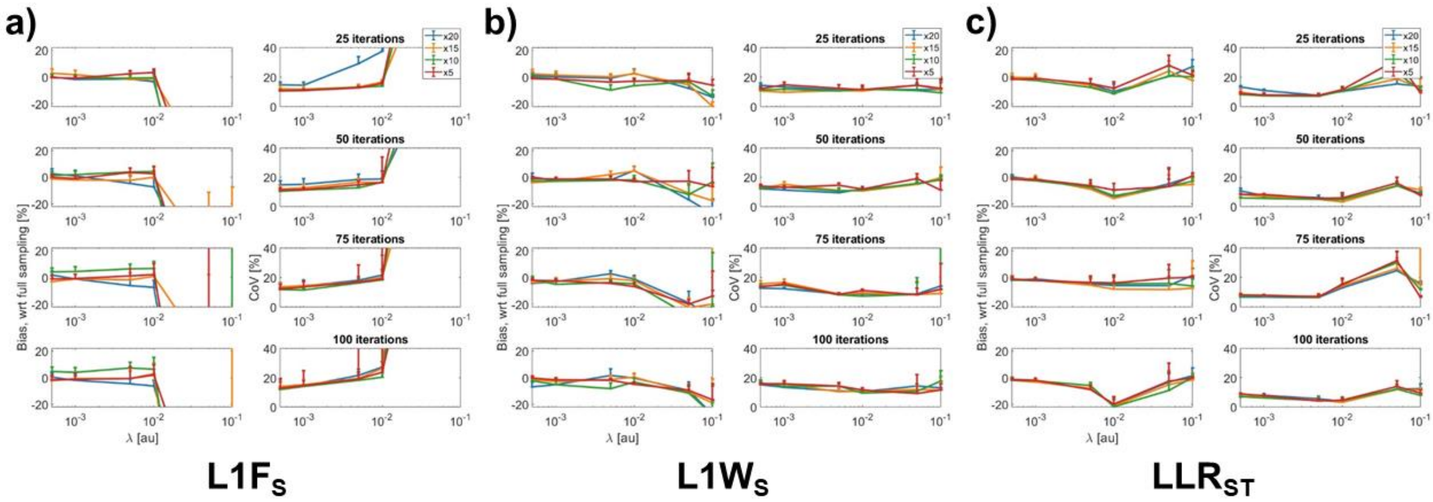

Figure S3: Bias and CoV as a function of regularization parameter across all undersampling factors. The panels present results for T1 maps calculated from images reconstructed using a) L1F$_S$, b) L1W$_S$, c) LLR$_{ST}$.

medRxiv preprint doi: https://doi.org/10.1101/2025.07.28.25332213; this version posted July 29, 2025. The copyright holder for this preprint (which was not certified by peer review) is the author/funder, who has granted medRxiv a license to display the preprint in perpetuity.
It is made available under a CC-BY 4.0 International license .

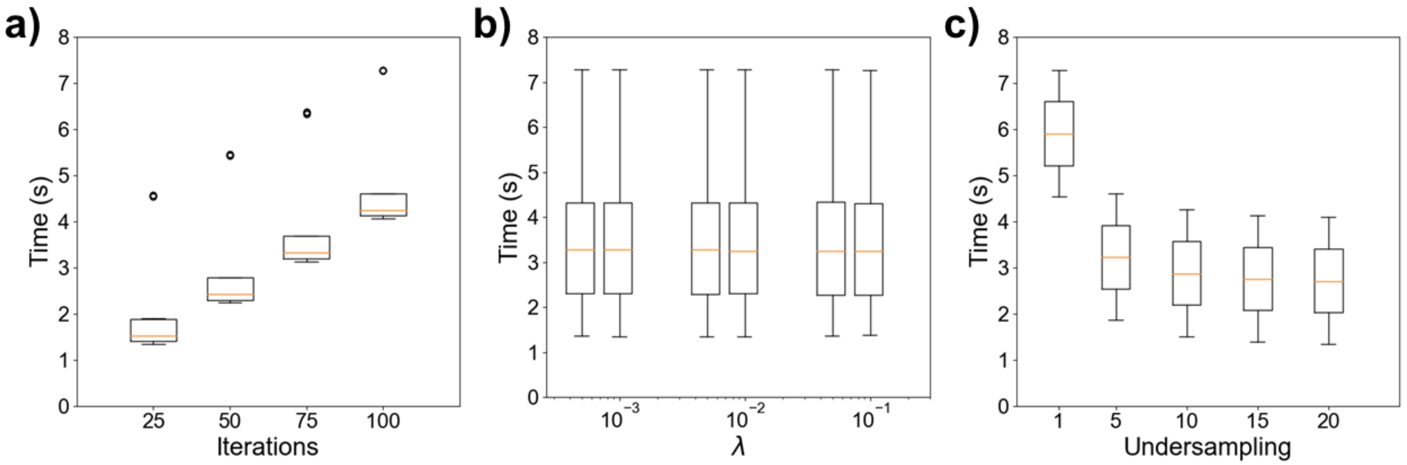

Figure S4: PICS reconstruction time across the number of iterations (a), regularization parameter (b), and undersampling factor (c). Note that the reported values do not include the calculation of the coil sensitivity maps, which in this case was on the order of 8 - 12min. Outlier in panel (a) represents reconstruction for fully sampled dataset.
